# Implants in Adolescents: Clinical Outcomes After Growth-Pattern-Adapted Implant Placement

**DOI:** 10.3390/jfb17070332

**Published:** 2026-07-09

**Authors:** Felix Tetsch, Milan Stoilov, Dominik Kraus, Michael Marder, Jan Tetsch, Norbert Enkling

**Affiliations:** 1 Department of Reconstructive Dentistry and Gerodontology, School of Dental Medicine, University of Bern, 3012 Bern, Switzerland; felix.tetsch@uk-muenster.de (F.T.); stoilov@uni-bonn.de (M.S.); dominik.kraus@ukbonn.de (D.K.); 2 Department of Cranio and Maxillofacial Surgery, University Hospital Münster, 48149 Münster, Germany; 3 Dr. Tetsch and Colleagues, 48151 Münster, Germany; dr.tetsch@t-online.de; 4 Department of Prosthodontics, Preclinical Education and Dental Materials Science, University Hospital Bonn, 53111 Bonn, Germany; michael.marder@ukbonn.de

**Keywords:** dental implants, dental implantation, maxillofacial development, adolescent, adolescent development, tooth loss, hypodontia

## Abstract

(1) Background: Dental implants in adolescents remain controversial. This retrospective study aimed to evaluate the esthetic outcome of implants placed during adolescence using a growth-pattern-adapted concept, with implant survival, peri-implant health, and patient satisfaction as secondary outcomes. (2) Materials and methods: Primary implant survival was retrospectively evaluated based on treatment records in 305 patients (612 implants) affected by tooth loss or congenital tooth agenesis. A subgroup of 68 anterior maxillary implants (50 patients, mean age at implantation: 16.2 ± 2.3 years; range: 12–20) were clinically re-examined after a mean follow-up of 92.4 ± 61.2 months (range: 36–288). (3) Results: Primary implant survival was 93.8% overall (tooth loss: 97.7%; tooth agenesis: 92.3%). The clinical follow-up cohort showed no cases of peri-implantitis and patient satisfaction was high. Anterior maxillary implants demonstrated favorable esthetic outcomes (Pink Esthetic Score [PES]: 12.0 ± 1.1), with females exhibiting significantly higher scores than males. Vertical discrepancies between the implant crown and the contralateral tooth were observed in 41.2% of implants (infraocclusion: 32.4%; supraocclusion: 8.8%). They were significantly associated with tooth loss, non-mesofacial growth patterns and younger age at implantation, without significant influence on PES. (4) Conclusions: The growth-pattern-adapted implant placement met the predefined esthetic threshold and resulted in high primary implant survival, stable peri-implant health, and high satisfaction, supporting its feasibility for anterior maxillary implant placement during adolescence.

## 1. Introduction

It is commonly known that osseointegrated dental implants placed in patients with pending growth do not participate in further maxillary growth and might lead to unsatisfying outcomes [1]. A sufficiently osseointegrated implant does not behave like a naturally erupting tooth, but rather like an ankylosed tooth [2,3]. This can particularly lead to esthetic and functional problems, especially in the anterior maxilla, such as infraocclusion—a reported negative outcome of dental implants in growing patients [4]. Implants do not adjust to the maxillomandibular growth like natural teeth do, which can lead to negative implant positions, especially in single-tooth implants, compared to neighboring teeth [5]. Thus, the current literature supports the use of dental implants in growing patients only under specific circumstances [6] and further recommends implant treatment deceleration in adolescents [7]. Consequently, dental implants in growing patients are commonly assumed to be risky, especially in the esthetic region. However, there is currently no scientific evidence for the ideal placement timing in implantology to avert infrapositioned implants in continuously developing patients [8]. Furthermore, it is reported that the available evidence-based literature is not sufficient to evaluate the usage of dental implants in children [9]. Therefore, the treatment of children and adolescents with dental implants remains a persisting controversial topic and needs further examination.

The vertical dimension of facial growth continues the longest—female vertical facial growth usually lasts until the age of 17 to 18 years and growth in males even tends to last longer, while also being affected by the growth type [10]. Vertical growth tendencies can be evaluated by orthodontic cephalometric analysis, allowing clinicians to assess individual growth patterns. Since natural teeth continue to erupt even later on in life, a defined age limit is said not to be a useful guideline in implantology [11]. Furthermore, it is reported that the dental arches continually transform in adulthood, even until the sixth decade, although the extent of transformation decreases with increasing age [12].

Prosthetic treatment indication in childhood or adolescence might become necessary because of tooth loss or congenital tooth agenesis and is often very challenging for clinicians. Tooth loss due to dental trauma has been reported as the most frequent cause for missing permanent teeth in growing patients (73.13%), followed by congenitally missing teeth (18.19%) [13]. Congenitally missing upper lateral incisors have been reported with a prevalence of 1.3% [14]. Kaba and Maréchaux [15] observed a prevalence of dental trauma to permanent teeth of approximately 11% in patients aged 6–18 years, while Hamilton et al. [16] even reported a prevalence of 34% (12% needing therapy) in pupils aged 11–14 years.

Following tooth loss, dentists commonly face a therapeutic problem: the alveolar process adapts and atrophies horizontally and vertically [17]. On average, an extraction socket takes twelve weeks to finally close and heal [18], while the alveolar bone atrophies vertically 11–22% and horizontally 29–63% in the first six months following tooth extraction; in the first 3–6 months particularly, bone loss occurs quickly [19].

Therefore, following tooth loss at a young age, clinicians face progressive bone atrophy and further eruption of the adjacent teeth may additionally increase the resulting tissue defects vertically. Thus, bone grafting is often necessary due to insufficient bone volume to facilitate successful implant placement [20]. Additionally, the consequences of dental trauma may lead to significant tissue deficits as well [21]. The alveolar process develops as a product of erupting teeth and their periodontal ligaments [22]. Therefore, patients with congenital tooth agenesis frequently display an underdeveloped hypoplastic alveolar process; usually, this does not undergo significant vertical atrophy in single-tooth gaps, where the adjacent teeth and/or the persisting deciduous tooth often preserve the dimension of the crestal alveolar bone.

The clinician’s treatment dilemma is a young patient with congenital tooth agenesis or (traumatic) tooth loss at an age at which dental implants are not a recommended treatment option, often resulting in a wait-and-see approach with provisional prosthetics. While removable prostheses are reported to be the most frequently used treatment option in growing patients, they are particularly associated with psychosocial and functional challenges in children [23]. Alternative treatment options include resin-bonded fixed dental prostheses (Maryland bridges), orthodontic space closure, and conventional fixed dental prostheses, which avoid the need for implant placement, as well as autotransplantation, which is limited by donor tooth availability. Survival rates of single-tooth implants in the anterior maxilla are reported to be up to 100% after ten years [24]. Contrarily, in patients with congenitally missing teeth, implant survival was observed to be reduced in children below the age of 13 years at 72.4% and slightly reduced in adolescents below the age of 18 years at 93.0%, compared to adults with 97.4% success rates [25]. However, implant survival alone does not equal a successful treatment. At present, factors such as esthetic outcome, peri-implant health and patient satisfaction should be considered to be predictable in standardized dental implant treatment.

Park et al. [26] reported that age alone does not seem to affect implant survival in elderly patients >65 years; Grant and Kraut [27] reported implant survival rates of 99% in the maxilla and 100% in the mandible in patients aged 79–99 years, highlighting the reliability of dental implants in geriatric dentistry. However, clear rules in young patients regarding definitive implant therapy do not seem to exist.

A concept of implant treatment in growing patients was described for the first time in Münster, Westphalia, over 25 years ago. By modifying the implant position according to the patient’s individual growth pattern, this concept aims to achieve esthetic symmetry once the main phase of facial growth has been completed [28].

The primary aim of this study was to evaluate the esthetic outcomes using the Pink Esthetic Score (PES); this is a validated tool for evaluating peri-implant soft tissue outcomes (cf. Section 2.4) of implants placed in adolescent patients according to the growth-pattern-adapted treatment concept. The secondary aims included the assessment of implant survival, peri-implant health, and patient-centered outcomes.

## 2. Materials and Methods

### 2.1. Study Design

This retrospective observational study is based on treatment records from a private practice (Dr. Tetsch & Colleagues, Münster, Germany) between 2000 and 2021. The follow-up was conducted between September 2024 and January 2025 to analyze the outcomes of dental implants placed during adolescence in patients aged between 12 and 20 years. The study was conducted in accordance with the Declaration of Helsinki—General Assembly of the World Medical Association [29]. Ethical approval was granted by the Ethics Committee of the Medical Faculty of the University of Bonn (CEC; No. 2024-331-BO).

In the first observational analysis, all patients who had received dental implants be- tween the ages of 12 and 20 years were evaluated for primary implant survival based on treatment documentation. In a second step, patients were invited for clinical follow-up based on predefined inclusion criteria (cf. below). Participants who presented for the clinical reevaluation provided written informed consent prior to study inclusion. The study was carried out at a private practice (Dr. Tetsch & Colleagues, Münster, Germany), in cooperation with the University of Bonn and University of Bern.

The aim of the first part of the study was to assess the primary implant failure rates in this age group and to compare outcomes between the indication subgroups tooth loss and congenital tooth agenesis, as well as to contextualize these findings alongside data from adult and adolescent populations. The second part of the study focused on evaluating esthetic outcomes in single-tooth implants placed in the anterior maxilla and identifying influencing factors, including age at implant placement, in situ duration, gender, treatment indication and facial growth type (mesofacial, brachiofacial, dolichofacial). In addition, peri-implant health was assessed. To evaluate patient satisfaction and patient-centered outcomes, a standardized questionnaire was conducted.

### 2.2. Growth-Pattern-Adapted Implant Placement (2000–2021)

Following an orthodontic cephalometric analysis, implants were placed with the aim of achieving symmetry to the contralateral tooth after most facial growth had been completed. Implant positioning was conducted following the guidelines for implant placement in the anterior maxilla, as defined by Buser et al. [30]. Since implantation, they were then prospectively adjusted based on patients’ individual growth patterns:

- The vestibulo-oral implant position was slightly alternated, with further palatal adjustment in patients with a clockwise growth pattern and further vestibular adjustment in patients with a counter-clockwise growth pattern.
- The insertion angle was set to be marginally steeper in patients with a clockwise growth pattern and shallower in patients with a counter-clockwise growth pattern.
- The apico-coronal implant position had been adjusted according to growth type (mesofacial, brachiofacial, dolichofacial) and the implants were generally placed more coronally; meanwhile, exposed sections of the implants were augmented with the GBR technique (autogenous and xenogenous grafting particles).

Due to the continued eruption of adjacent teeth, the incisal edge of the implant crown might appear shortened over time. Therefore, patients were informed in advance about the potential need for future adjustments or replacement of the prosthetic restoration [28].

### 2.3. Selection of Participants and Eligibility Criteria

Retrospective analysis:

All patients who received implants between the ages of 12 and 20 years according to the growth-pattern-adapted protocol were retrospectively screened for primary implant survival based on treatment documentation. No exclusion criteria were applied.

Clinical follow-up cohort:

Patients who had been treated with single-tooth bone level titanium implants and definitive single crowns in the anterior maxilla (regions 13 to 23) between the ages of 12 and 20 years according to the protocol were selected for re-examination.

The additional selection criteria were as follows:

- A maximum of two implants in the anterior maxilla (regions 13 to 23);
- At least three years since implant placement;
- A minimum age of 18 years at the time of study participation/recall.

Patients were excluded if any of the following criteria applied:

- Age under 18 years at the time of study participation;
- Lack of written informed consent;
- Inability to understand the scope, significance, and consequences of the study;
- Inability to follow the study procedure;
- Known or ongoing abuse of medication, drugs, or alcohol.

### 2.4. Clinical Data Collection and Evaluation

All clinical follow-up examinations were conducted under standardized conditions in the same clinical setting to ensure consistency and comparability across all assessments. Each recalled patient underwent a structured protocol that included photographic documentation, a digital intraoral scan, peri-implant health examination and a standardized questionnaire.

Photographic Documentation:

Frontal and upper-frontal photographs were captured using a standardized digital camera (Canon EOS 7D Mark II; Canon ©, Tokyo, Japan), both with and without cheek retractors.

These images were used to assess soft tissue esthetics and for visual documentation.

Digital Intraoral Scanning:

Intraoral scans of both the upper and lower jaws were performed using the same device for all patients (Trios 5; 3Shape ©, Copenhagen, Denmark). These scans were also used for the esthetic evaluation, as well as the digital analysis of crown position and vertical discrepancies.

Esthetic Evaluation:

The esthetic outcome of each implant site was assessed using both photographic images and intraoral scans. The Pink Esthetic Score [31] was used to evaluate soft tissue esthetics around the implant restorations. The PES consists of seven soft tissue parameters, each of which can be rated on a scale from 0 to 2. The maximum total score is 14, with higher scores indicating more favorable esthetic outcomes. The PES parameters are defined as follows in Table 1.

**Figure 1:**
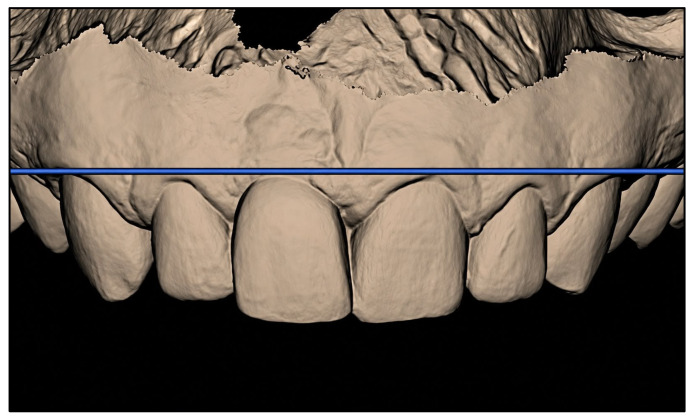
Schematic depiction of infra- and supraocclusion determination. The implant crown [position 11] shows no vertical difference (Difference: 0 mm)) when compared to the contralateral tooth [position 21]. The blue horizontal line represents the intercanine marginal line, which was used as the reference for assessing vertical discrepancies.

Vertical alignment and discrepancies, defined as infra- or supraocclusion of the implant crown relative to the contralateral tooth, were digitally measured using the intercanine marginal line as a reference (Figure 1).

The assessment was independently performed by three experienced clinicians specializing in implant dentistry and oral surgery.

Assessment of Peri-Implant Health:

Peri-implant health was clinically evaluated by the same examiner in all cases, following the diagnostic criteria established by the 2017 World Workshop on the Classification of Periodontal and Peri-Implant Diseases and Conditions [32].

Applied criteria:

- Presence of bleeding and/or suppuration on gentle probing;
- Probing depths of ≥6 mm;
- Bone levels ≥3 mm apical of the most coronal portion of the intraosseous part of the implant, as evaluated by sounding the implant shoulder and the crestal bone.

Patient-Reported Outcomes:

To assess the patients’ subjective experience, a standardized questionnaire was handed out. It included seven questions covering esthetic, functional and psychosocial aspects of the implant treatment.

Each item was rated on a 5-point Likert scale (1 = best, 5 = worst):

1. How much did the tooth gap bother you before the treatment?
2. How satisfied were you with the temporary prosthetics (fixed or removable)?
3. How much did the implant surgery stress you?
4. How much pain did you experience after the surgery?
5. How satisfied are you with the final implant crown?
6. Can you bite and chew normally with the implant crown?
7. Would you choose the implant treatment again?

### 2.5. Statistical Analysis

Analyses were conducted using data from participants with available follow-up information. Data collection was performed by a single clinician. A database comprising 50 patients with a total of 68 dental implants was created using “Excel” (version number: 16.78.3, Microsoft © Corporation, Redmond, WA, USA). The collected data were analyzed using SPSS (Statistical Package for the Social Sciences, Version: 29, IBM, Armonk, NY, USA). Mixed models were fitted using restricted maximum likelihood (REML) estimations and the Kenward–Roger approximation for small sample size was used. Data are presented as mean ± SD or mean [95% CI]. The level of significance was set at *p* = 0.05. The main objective of this study was to compare the esthetic outcome of implants placed in adolescent patients according to the growth-pattern-adapted treatment concept to commonly established standards in adult patients. The following null hypothesis (H_0_) was tested: The applied growth-adapted treatment concept for implant placement in adolescents does not result in an acceptable Pink Esthetic Score (more than 5% of implants with PES < 8) in the follow-up cohort.

Furthermore, influencing factors on implant-related outcomes were examined in an exploratory approach. Implant survival rates (I), peri-implant health (II) and self-reported patient satisfaction (III) were descriptively evaluated. The Pink Esthetic Score (PES) was independently assessed for each implant by three raters, all of whom are clinicians specialized in implant dentistry and oral surgery. Prior to the assessment, a calibration session was conducted using reference images to ensure consistent application of the PES criteria. Inter-rater reliability was high, with a Cronbach’s alpha of 0.882 [95% CI: 0.823–0.923].

To determine whether the treatment concept yields acceptable results (PES not lower than 8), we determined whether more than 95% of cases can be expected to have PES values > 8. PES values were transformed by subtracting 8 PES + 2 SD from each observation (1 SD = 0.94 PES at patient level + 0.58 residual PES = 1.1 PES). The transformed variable was used for hypothesis testing.

A linear mixed-effects model was fitted with implants nested within patients and patient ID was included as a random effect. Non-inferiority was concluded if the estimated intercept of the null model was significantly greater than zero. Clustering was confirmed, with 72% of observed variation in PES attributable to the patient level. To assess the influence of the patient age at implant placement [years], implant duration in situ [months], patient sex, and treatment indication factors on the PES, as well as on vertical discrepancies, a linear mixed-effects model was used, each with patient ID as random factor. Facial growth type could not be included as a factor, because of the low frequencies of two categories in the sample. To test the influence of predictive factors on vertical discrepancies, a multilevel binary logistic regression model with presence of vertical discrepancy (binarized) as the outcome variable was fit, with patient ID as random effect. Patient sex, treatment indication and age at implantation [binarized at 15.5 years; ROC-analysis] were included as fixed factors. As for the other models, facial growth type could not be included as a factor due to the small sample size. The association with vertical discrepancies was therefore descriptively analyzed with a Fisher’s exact test.

## 3. Results

### 3.1. Study Population

The initial patient pool for the retrospective analysis comprised 305 patients (148 female, 157 male), who had received a total of 612 implants placed between the ages of 12 and 20 years. Among these, 441 implants were placed for the indication of congenital tooth agenesis and 171 due to tooth loss. Focusing on the esthetic region of the anterior maxilla, 238 patients (116 female, 122 male) received a total of 367 implants. After applying the aforementioned inclusion criteria, a final cohort of 139 patients (63 female, 76 male) with 173 implants remained eligible for evaluation. Of these, 123 patients could successfully be contacted. Most had been referred for implant treatment and continued follow-up with local dentists. A total of 36 patients declined participation due to time constraints or lack of interest, though they reported subjective satisfaction with their implants. A total of 34 patients were unable to participate due to distant residence but also all reported subjective satisfaction. Two patients did not show up for the appointment multiple times. One patient was reported deceased.

A total of 50 patients (22 female, 28 male) with 68 anterior maxillary implants were successfully called in for the follow-up examination. Of the 68 implants, 38 were placed in male patients and 30 in female patients. The indications for implant treatment were tooth loss (*n* = 38) and congenital tooth agenesis (*n* = 30). The mean age at the time of implant placement was 16.2 ± 2.3 years (median: 17; range: 12–20). At the time of follow-up, the implants had been in situ for a mean duration of 92.4 ± 61.2 months (median: 68.5; range: 36–288). Bone augmentation was performed in all cases with autogenous and xenogenous grafting material, either simultaneously with implant placement or in a separate preliminary procedure.

### 3.2. Growth-Pattern-Adapted Implant Positioning—Representative Cases

Here, we describe a clinical case of implant positioning in regio 11 after traumatic tooth loss in a 12-year-old female patient with a mesofacial growth type and a moderately counter-clockwise rotational tendency of the maxillo–mandibular complex.

**Figure 2:**
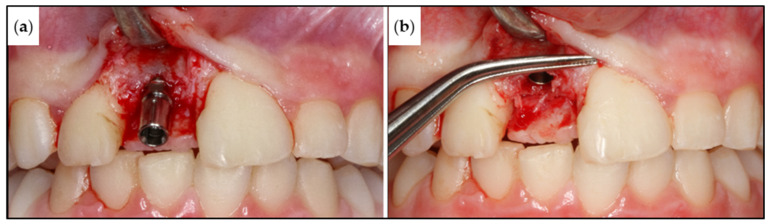
(**a**) Implant site preparation and growth-pattern-adapted positioning at implant surgery. Implant placed slightly vestibular, with shallower angulation and a more coronal placement. (**b**) Definitive implant position after insertion.

**Figure 3:**
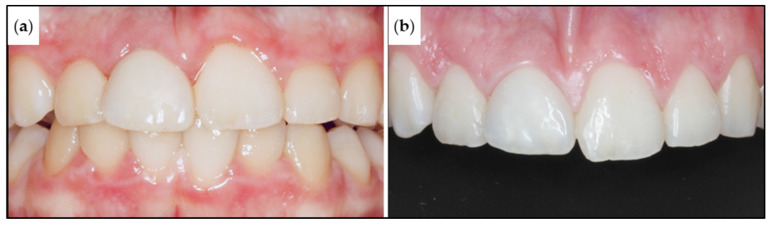
(**a**) Clinical situation six months after surgery, showing excess of marginal soft tissue at the implant crown at position 11. (**b**) Two years postoperatively, an alignment of the emergence profile can be observed, whereas the shortened incisal edge of the first crown is clearly apparent.

**Figure 4:**
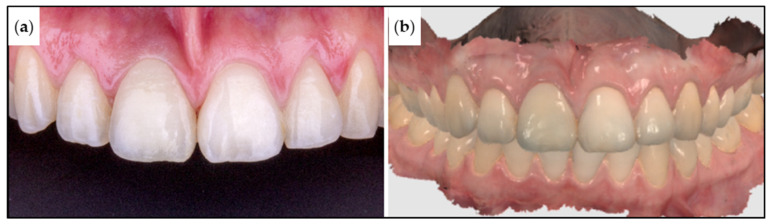
(**a**) Seven years after surgery (patient’s age: 19 years), a new implant crown exhibits both marginal tissue alignment and leveled incisal edges. (**b**) Intraoral scan.

At implant surgery, a mucoperiosteal flap was raised, the implant site was prepared, and the implant was positioned in a slightly modified position. The implant (Conelog^®^ ScrewLine 3.3 × 13.0 mm; Camlog ©, Wimsheim, Germany) was positioned slightly vestibularly, angulated shallower, and placed in a more coronal position than usual [Figure 2]. Exposed sections and the alveolar process contour had been augmented using autogenous bone chips and a xenogenous grafting material (Bio-Oss^®^; Geistlich © Pharma AG, Wolhusen, Switzerland). Six months after surgery, the first implant crown was still showing an overplus of soft tissue on the marginal site [Figure 3a], and two years postoperatively marginal changes had become visible. The incisal edge of the implant crown had shortened, whereas an alignment of the emergence profiles had occurred [Figure 3b]. Seven years after surgery (patient’s age: 19 years), a new implant crown had been placed and documented, showing marginal alignment as well as leveled incisal edges [Figure 4].

**Figure 5:**
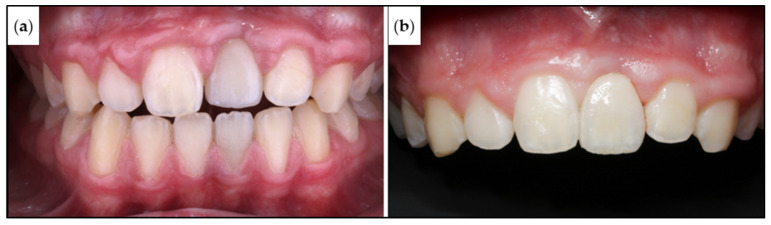
(**a**) Clinical situation in a male patient (age: 18 years) who had received a dental implant [position 21] at the age of 14 years. The first implant crown exhibited a palatal position as well as a shortened incisal edge due to craniofacial growth. (**b**) Despite infraocclusion, the remaining marginal soft tissue still allowed modification and further alignment of the emergence profile with a new implant crown.

The second case illustrates the clinical situation in a male patient (age: 18 years) who received a dental implant (Conelog^®^ ScrewLine 3.8 × 11.0 mm; Camlog ©, Wimsheim, Germany) in regio 21 at the age of 14. As a result of craniofacial growth, the implant crown displayed a palatal position and the incisal edge appeared shortened when compared to the contralateral tooth. The crown presented with infraocclusion, while an excess of soft tissue had remained at the marginal site [Figure 5a]. This allowed for modification of the emergence profile and a prosthetically corrected position of the renewed implant crown [Figure 5b].

### 3.3. Retrospective Analysis—Cumulative Implant Survival

Of the initial 612 implants placed, 38 failures were recorded, resulting in a cumulative primary survival rate of 93.8%. Implants placed for the indication of congenital tooth agenesis showed a survival rate of 92.3% (407 out of 441), while implants placed due to tooth loss exhibited a higher survival rate of 97.7% (167 out of 171). All 38 failed implants lacked primary osseointegration and were therefore classified as early losses. They were removed during the uncovering surgery, followed by a reimplantation approximately twelve weeks later. The secondary interventions had resulted in a 100% implant survival rate. In the final patient pool, no implant loss was observed after a mean follow-up duration of 92.4 ± 61.2 months (median: 68.5; range: 36–288).

### 3.4. Clinical Follow-Up Cohort—Patient Characteristics

The clinical characteristics of the study cohort are summarized in Table 2.

### 3.5. Esthetic Outcomes

Pink Esthetic Score

The mean Pink Esthetic Score (PES) across all 68 implants was 12.0 ± 1.1. The median score was 12, with a range from 8.3 to 14.

A total of 42 implants (61.7%) achieved a PES of 12 or higher. Only three implants (4.4%) received PESs below ten. Of the 68 evaluated implants, three implants placed in three male patients received PESs below ten. These cases are descriptively analyzed in greater detail to identify potential risk factors. The first implant, with a PES of 9.7, was placed in regio 11 due to tooth loss. This patient previously received two implants—regions 11 and 12—inserted alio loco. One of them—implant 11—was removed due to peri-implant infection and associated bone loss. A second implant was placed at the age of 20, three years after the first implantation. The adjacent implant was placed at 17 years according to standard positioning, exhibited slight misalignment. The second implant, which scored 8.3, was placed in a patient who had lost the tooth at the age of 9 years. The implant was placed at age 13, following a more extensive two-staged bone augmentation. Additionally, the patient presented with a pronounced dolichofacial growth pattern. The third implant, scoring 8.7, was placed at the age of 17, following tooth loss at the age of 14 years and an extended edentulous period.

No further relevant risk factors could be identified in these patients, and similar risk factors were also present in other cases with higher PES outcomes. Therefore, these three implants were not excluded from the analysis. The mean values for each PES parameter are presented in Table 3.

The mean Pink Esthetic Score (PES) for implants placed in female patients (*n* = 30) was 12.44 [95% CI: 12.01–12.87], compared to 11.71 [95% CI: 11.28–12.14] in male patients (*n* = 38). The difference between male and female patients was statistically significant (Diff: 0.73 PES [0.12 to 1.35]; F (1,41) = 5.771; *p* = 0.021), with higher PESs observed in female patients. No statistically significant difference in PES between implants placed in patients with congenital tooth agenesis and implants placed following tooth loss was detected.

No statistically significant influence of age at implant placement on the Pink Esthetic Score (PES) was detected. There was a non-statistically significant tendency for lower Pink Esthetic Scores (PESs) in implants with longer in situ duration (F (1,42) = 3.464; *p* = 0.070). Overall, the mean PES decreased by 0.054 for every additional year in situ ([95% CI: 0.113 to −0.005]; *p* = 0.07).

Vertical discrepancies

A total of 28 implants (41.2%) showed a measurable vertical discrepancy in comparison to the contralateral tooth. Among these, 22 implant crowns (32.4%) were in infraocclusion, while 6 implant crowns (8.8%) showed a supraocclusion with regard to the gingival margin of the contralateral tooth. A total of 40 implant crowns (58.8%) exhibited no measurable vertical deviation. The mean vertical difference of the implants standing in infraocclusion (*n* = 22) was 0.8 ± 0.5 mm (median: 0.7; range: 0.02 to 2.06). The mean vertical difference of the implants standing in supraocclusion (*n* = 6) was 0.8 ± 0.5 mm (median: 0.8; range: 0.2 to 1.7). Of the 68 evaluated implants, 7 cases (10.3%) presented with a vertical difference of ≥1.0 mm between the implant crown and the contralateral tooth (four male and three female patients). The predominant indication in this subgroup was tooth loss (*n* = 6).

The mean age at the time of implant placement in these patients was 14.7 ± 2.7 years (median: 14; range: 12–20). The mean vertical deviation was −1.1 ± 1.2 mm (median: 1.4; range: −2.06 to +1.7). The occurrence of vertical discrepancies compared with the contralateral tooth was associated with implant indication, age at the time of implant placement and facial growth type. Among implants placed in patients with congenital tooth agenesis, 11% [95% CI: 2–18%] showed vertical differences, compared to 82% [95% CI: 58–94%] of the implants in patients with tooth loss. The difference was highly statistically significant (odds ratio: 38.5 [95% CI: 3.65–500]; *p* = 0.002). Of the implants placed in patients younger than 15.5 years of age, 76% [95% CI: 45–92%] showed a vertical difference, compared to 15% [95% CI: 4–40%] of the implants placed in patients older than 15.5 years. This difference was also statistically significant (odds ratio: 18.5 [95% CI: 2.1–160]; *p* = 0.008). Regarding facial growth patterns, the study cohort included 39 patients with a mesofacial, 8 patients with a dolichofacial (long face) and 3 patients with a brachiofacial (short face) growth pattern. The vertical discrepancies in implants compared to the contralateral tooth according to each growth pattern were as follows:

- Mesofacial (*n* = 55): mean vertical difference: 0.2 ± 0.3 mm (median: 0.0; range: 0–1.22).
- Brachiofacial (*n* = 3): mean vertical difference: 1.1 ± 0.5 mm (median: 0.9; range: 0.61–1.7).
- Dolichofacial (*n* = 10): mean vertical difference: 1.0 ± 0.7 mm (median: 0.8; range: 0–2.06).

Due to the small sample sizes of non-mesofacial growth types, statistical analysis was limited. However, vertical discrepancies were observed in 90.9% of the patients with non-mesofacial growth patterns, compared to only 41% among those with a mesofacial growth pattern. The difference between these groups was statistically significant (Fisher’s exact test: *p* = 0.005).

### 3.6. Peri-Implant Health Results

One female patient presented with bleeding on probing, which was attributed to a loosened implant crown. Following reattachment of the crown, the bleeding had fully resolved at a follow-up visit three weeks later. None of the patients exhibited probing depths of ≥6 mm, nor was bone loss beyond 3 mm observed.

### 3.7. Patient-Reported Outcomes

Before treatment, most patients perceived the existing tooth gap to be highly stressful, with a mean score of 4.1 ± 1.1 (median: 4; range 1–5). Among all patients, 82% rated this question with a score of four or five. Satisfaction with the temporary prosthetic restoration was moderate. Among the 17 patients who received a fixed provisional, the mean rating was 3.1 ± 1.1 (median: 3; range: 1–5), while the 24 patients treated with removable provisionals rated theirs slightly lower at 3.4 ± 1.3 (median 3; range: 1–5). Nine patients had not received temporary restorations and therefore did not rate this question. Surgical stress was generally assessed as low, reflected by a mean score of 1.7 ± 0.8 (median: 2; range 1–4), with 86% indicating minimal strain (scores one or two). Postoperative pain was also predominantly mild, indicated by a mean score of 2.0 ± 1.0 (median: 2; range: 1–5), with nearly three quarters reporting only slight discomfort (scores one or two). The final implant crown received a mean score of 1.1 ± 0.3 (median: 1; range: 1–2). A total of 43 patients (86.0%) rated this question with a score of one. The ability to bite and chew normally was rated at a mean of 1.1 ± 0.2 (median: 1; range: 1–2). A total of 47 patients (94.0%) reported best function (score = 1). Overall satisfaction with the treatment was rated with a mean score of 1.1 ± 0.2 (median: 1; range: 1–2). A total of 47 patients (94.0%) rated the question “Would you choose implant treatment again?” with the highest possible score.

## 4. Discussion

In this cohort of implants placed during adolescence using a growth-pattern-adapted treatment concept, esthetic outcomes were favorable—with a mean PES of 12.0 ± 1.1 and no implant falling below the predefined esthetic threshold of PES = 8—peri-implant health was stable with no cases of peri-implantitis detected, and patient satisfaction was high, with 94% of patients indicating they would choose implant therapy again, after a mean follow-up of more than seven years. Vertical discrepancies between implant crowns and the contralateral tooth were observed in 41.2% of implants; however, the magnitude of these discrepancies was generally small (mean infraocclusion: 0.8 ± 0.5 mm; mean supraocclusion: 0.8 ± 0.5 mm). They were significantly associated with non-mesofacial growth patterns, younger age at implantation and the indication of tooth loss. Primary implant survival in the retrospective analysis was higher for implants placed due to tooth loss than for those placed in cases of congenital tooth agenesis.

The higher survival rate (97.7%) is in line with the findings by Gotfredsen [24], who reported a 100% survival rate after 10 years in early and delayed placed single-tooth implants in the anterior maxilla in patients aged 18–59 years. Similarly, Bonfante et al. [33] reported a 100% implant survival rate in 21 patients aged 14–19 (predominantly 16–18) years at the time of implant placement, based on 37 implants, after a mean follow-up duration of 10 years. Terheyden and Wüsthoff [25] reported significantly reduced implant survival rates in patients with congenital tooth agenesis below the age of 13 (72.4%) and slightly reduced survival in patients under 18 years (93.0%), compared to adults (97.4%). These findings are consistent with the observations of the present study, which showed a slightly reduced primary survival rate of 92.3% for implants placed due to congenital tooth agenesis. A narrow cortical alveolar ridge with clinically reduced vascularization might contribute to the slightly increased risk of implant loss in patients with congenital tooth agenesis.

The mean Pink Esthetic Score (PES) of 12.0 ± 1.1 after a mean implant in situ duration of over seven years is comparable to or even slightly higher than values reported in adult populations. Rokn et al. [34] reported a mean baseline PES of 11.63 (SD 1.61; range 7–14) and a long-term PES after 10–12 years in situ of 11.05 (SD 2.09; range 6–14) in adult patients (mean age: 43.5 years). To the best of the authors’ knowledge, there are currently no published studies assessing PES in a comparable adolescent cohort with a similarly long follow-up, thereby limiting direct comparison. The significantly higher PES observed in female compared to male patients (*p* = 0.021) may be explained by the generally earlier physical maturation in females, with peak height velocity typically occurring at around age 11.5 in girls, compared to 13.5 in boys [35]. However, three male patients exhibited PESs below ten, potentially confounding the comparison between male and female outcomes. To validate these findings, further studies with larger cohorts are needed. The non-significant tendency toward lower PESs for implants with longer in situ durations is in line with the findings shared by Rokn et al. [34] in adult populations. The findings indicate that implants in the esthetic zone of the anterior maxilla during adolescence, using the aforementioned positioning concept, can lead to esthetic and functional outcomes comparable to those observed in adult implantology.

The lower rate of vertical discrepancies in implants placed due to congenital tooth agenesis compared to tooth loss (*p* = 0.002) may be attributed to the overall more controlled and long-term planned clinical setting in agenesis cases, compared to cases of traumatic tooth loss—including preliminary orthodontic treatment commonly required in agenesis cases to create sufficient coronal and apical space for implant placement [36]. Furthermore, cases of dento-alveolar trauma may result in long-term damage to both hard and soft tissues [21], placing the clinician at a physical and temporal disadvantage at the outset of treatment. Notably, these vertical discrepancies did not translate into lower PESs in the tooth loss group. Vertical discrepancies were significantly associated with non-mesofacial growth patterns (*p* = 0.005), with a tendency toward infraocclusion in dolichofacial patients, consistent with [28]. Given the small subgroup sizes, these findings should be interpreted with caution. A higher occurrence of vertical discrepancies was also observed in patients younger than 15.5 years at implant placement (*p* = 0.008), without concomitant deterioration of soft tissue esthetics (PES). It has been reported that the risk of passive tooth eruption appears to be lower in patients over 15 years of age [6], which is consistent with the findings of this study. However, the magnitude of infraocclusion observed in this study was lower than reported by Brugnolo et al. [37], who documented infraocclusion (up to 3 mm) in three examined patients aged 11.5–13 years after a follow-up period of 2.5–4.5 years. Infraocclusion was observed in the evaluated study population, but to a smaller extent [0.8 ± 0.5 mm (median: 0.7; range: 0.02–2.06 mm)] and at a lower frequency (32.4%). According to Agarwal et al. [10], vertical facial growth is almost complete by the age of 17–18 years, typically earlier in females, but additionally influenced by the individual facial growth pattern. Despite implant placement occurring before this growth completion in parts of the cohort, age at implant placement had no significant influence on the PES, suggesting that implants placed during adolescence using this positioning concept can achieve favorable esthetic outcomes. However, individual physical maturation should always be considered alongside chronological age when determining the appropriate timing for implant placement. In this context, Wittneben et al. [38] reported an ongoing vertical development of adjacent teeth next to single-tooth implants even in adults. Padovezi et al. [39] identified an incidence rate of 19.6% in incisal-level alterations between the adjacent tooth and single central maxillary incisor implants, which was not influenced by sex or age group. Furthermore, it has been reported that facial growth and tooth eruption commonly keep on until the third and possibly even the fifth decade of life, concluding that a delay of anterior maxillary implants is advisable [7]. Cocchetto et al. [40] assumed a high prevalence of infrapositioned dental implants in the anterior maxilla and reported that the majority of patients were either unaware of the discrepancy or, if aware, largely unconcerned about it. Sauvin et al. [41] reported a mean infraocclusion of 0.62 mm (range: 0.15–1.63 mm) in 23 adult patients with a mean age of 47.8 years (range: 18.9–65.8 years) at the time of implant crown insertion, demonstrating infraocclusion levels even in adults comparable to the findings observed in the present study. Among the 23 patients, eight were aware of the infraocclusion, though only four of them wished for a correction. In line with this, the seven patients in the present study with a vertical discrepancy of ≥1 mm reported overall high treatment satisfaction.

Overall, the esthetic outcomes observed in this adolescent cohort match or even exceed those reported in adult populations, underlining the potential of this growth-pattern-adapted implant placement in adolescent patients as a reliable long-term treatment approach. The consistently high Pink Esthetic Scores and low vertical discrepancies of the implant crowns, even in patients as young as 12 years at the time of implant placement, suggest that the pending horizontal and vertical facial growth may be adequately anticipated.

Peri-implant health remained stable in all 68 evaluated cases, with no probing depths ≥ 6 mm or crestal bone loss ≥ 3 mm [32]. One case of bleeding on probing was observed, which resolved quickly after the reattachment of a loosened implant crown. These findings suggest that, with appropriate oral hygiene and follow-up care, long-term peri-implant stability can be expected in adolescent implant patients.

The high levels of satisfaction observed regarding esthetics, functional outcomes and the overall treatment experience are consistent with previous reports using standardized PROMs (Patient-Reported Outcome Measures) among single-tooth implant patients [42,43]. In contrast with the findings of the present study, Al Najam et al. [44] observed significantly higher patient satisfaction in patients implanted due to congenital tooth agenesis, compared to those with implants placed after tooth loss. Especially in a young population, where psychosocial impact and esthetic demands are typically elevated, these results highlight the value of a carefully planned, individualized implant treatment strategy that avoids prolonged provisional treatment phases during this critical development period in life.

Based on the findings of this study, the evaluated concept appears to be applicable across a broad range of adolescent patients with tooth loss or congenital tooth agenesis. Favorable esthetic outcomes (PES) were observed across all implantation ages evaluated (12–20 years). Regarding vertical discrepancies, a mesofacial growth pattern, an age at implantation of >15.5 years and the indication of congenital tooth agenesis were associated with significantly lower rates. From a clinical perspective, and based on the authors’ experience, completed dental eruption and adequate oral hygiene compliance should additionally be considered when selecting appropriate candidates. These latter recommendations are based on clinical expertise rather than the present study data.

This study has several limitations. The retrospective design limits causal interpretation and the absence of a control group precludes direct comparison with established treatment concepts in adults. While the sample size of this study is adequate for general conclusions, relevant subgroup analyses (e.g., growth type) were apparently underpowered. Furthermore, clinical follow-up was not performed for all 305 patients included in the retrospective primary survival analysis. The clinical re-examination was intentionally restricted to implants in the anterior maxilla (regions 13–23), as this region is of primary relevance for esthetic outcome assessment—the main objective of this study. Implants placed in other regions were therefore not clinically re-examined as part of the study protocol. Additionally, the study was conducted in a single specialized clinical setting and a considerable proportion of non-responders from the initial cohort were unavailable for follow-up, so the analysis is based on observed cases rather than an intention-to-treat approach, which may limit generalizability. Further prospective, long-term studies with larger cohorts, adult control groups and standardized PROMs are required to confirm and generalize these findings.

## 5. Conclusions

The concept of growth-pattern-adapted implant placement supports favorable long-term esthetic, peri-implant and patient-reported outcomes in adolescent patients requiring anterior maxillary implants. The predefined esthetic threshold was met and implant survival rates were high, with differences observed between indications.

While primary implant survival was higher in patients treated for tooth loss (97.7%) than in those with congenital tooth agenesis (92.3%), the overall survival rate of 93.8% appears broadly coherent with long-term survival trends reported in both adult and adolescent cohorts.

The consistently high Pink Esthetic Scores were comparable to those reported in adult populations. No significant association between age at implantation (range: 12–20 years) or treatment indication and the PES was detected in this cohort, though this should be interpreted with caution given the limited sample sizes. Female patients exhibited significantly higher PESs than male patients.

Vertical discrepancies between implant crowns and the contralateral teeth were significantly associated with younger age at implantation (<15.5 years), the indication of tooth loss as well as non-mesofacial growth patterns. Nonetheless, overall soft tissue esthetics (PES) remained uncompromised.

No signs of peri-implant disease were detected based on established diagnostic criteria and patient-reported outcomes showed consistently high levels of satisfaction with esthetics, function and the overall treatment experience.

These findings suggest that careful, individualized planning according to the patient’s growth pattern may support favorable outcomes in adolescent implant placement; however, prospective studies with larger cohorts and control groups are needed to confirm these observations.

## Figures and Tables

**Table 1:** PES parameters and scoring. Each parameter is rated on a scale from 0 to 2. Higher scores indicate higher esthetic outcomes.

Parameter	0	1	2
1. Mesial papilla	Missing	Incomplete	Complete
2. Distal papilla	Missing	Incomplete	Complete
3. Tissue contours	Unnatural	Virtually natural	Natural
4. Gingival level	>2 mm	1–2 mm	<1 mm
5. Alveolar process	Clearly resorbed	Slightly resorbed	No difference
6. Coloring	Clear difference	Slight difference	No difference
7. Texture	Clear difference	Slight difference	No difference

**Table 2:** Patient characteristics of the follow-up cohort.

Parameter	Value
Number of patients [*n*]	50
–Female	22
–Male	28
Number of implants [*n*]	68
–In female patients	30
–In male patients	38
Mean age at implantation [yrs ± SD]	16.2 ± 2.3
Median age at implantation [yrs (range)]	17 (12–20)
Mean follow-up duration [mo ± SD]	92.4 ± 61.2
Median follow-up duration [mo (range)]	68.5 (36–288)
Indication for implantation [*n*]	68
–Traumatic tooth loss	38
–Congenital tooth agenesis	30

**Table 3:** Observed mean scores and measures of dispersion for each PES parameter in the follow-up cohort.

PES Parameter	Mean ± SD (Median; Range)
Mesial papilla	1.8 ± 0.4 (median: 2; range: 0–2)
Distal papilla	1.8 ± 0.5 (median: 2; range: 0–2)
Tissue contours	1.9 ± 0.4 (median: 2; range: 1–2)
Gingival level	1.9 ± 0.4 (median: 2; range: 0–2)
Alveolar process	1.3 ± 0.5 (median: 1; range: 0–2)
Coloring	1.5 ± 0.6 (median: 2; range: 0–2)
Texture	1.8 ± 0.4 (median: 2; range: 1–2)

## Data Availability

The original contributions presented in this study are included in the article. Further inquiries can be directed to the corresponding author.

## References

[1] Shah R.A., Mitra D.K., Rodrigues S.V., Pathare P.N., Podar R.S., Vijayakar H.N. (2013). Implants in adolescents. J. Indian Soc. Periodontol..

[2] Odman J., Grondahl K., Lekholm U., Thilander B. (1991). The effect of osseointegrated implants on the dento-alveolar development. A clinical and radiographic study in growing pigs. Eur. J. Orthod..

[3] Thilander B., Odman J., Grondahl K., Lekholm U. (1992). Aspects on osseointegrated implants inserted in growing jaws. A biometric and radiographic study in the young pig. Eur. J. Orthod..

[4] Kamatham R., Avisa P., Vinnakota D.N., Nuvvula S. (2019). Adverse Effects of Implants in Children and Adolescents: A Systematic Review. J. Clin. Pediatr. Dent..

[5] Oesterle L.J., Cronin R.J. (2000). Adult growth, aging, and the single-tooth implant. Int. J. Oral Maxillofac. Implants.

[6] Bohner L., Hanisch M., Kleinheinz J., Jung S. (2019). Dental implants in growing patients: A systematic review. Br. J. Oral Maxillofac. Surg..

[7] Mijiritsky E., Badran M., Kleinman S., Manor Y., Peleg O. (2020). Continuous tooth eruption adjacent to single-implant restorations in the anterior maxilla: Aetiology, mechanism and outcomes—A review of the literature. Int. Dent. J..

[8] Klinge A., Tranaeus S., Becktor J., Winitsky N., Naimi-Akbar A. (2021). The risk for infraposition of dental implants and ankylosed teeth in the anterior maxilla related to craniofacial growth, a systematic review. Acta Odontol. Scand..

[9] Cherian J.M., Samuel S., Sabu A.M., Thomas A.M., Injety R.J. (2023). Dental implants in growing patients: A quality assessment of systematic reviews. J. Oral Biol. Craniofac. Res..

[10] Agarwal N., Kumar D., Anand A., Bahetwar S.K. (2016). Dental implants in children: A multidisciplinary perspective for long-term success. Natl. J. Maxillofac. Surg..

[11] Thilander B., Odman J., Jemt T. (1999). Single implants in the upper incisor region and their relationship to the adjacent teeth. An 8-year follow-up study. Clin. Oral Implants Res..

[12] Dager M.M., McNamara J.A., Baccetti T., Franchi L. (2008). Aging in the craniofacial complex. Angle Orthod..

[13] Elagib M.F.A., Alqaysi M.A.H., Almushayt M.O.S., Nagate R.R., Gokhale S.T., Chaturvedi S. (2023). Dental implants in growing patients: A systematic review and meta-analysis. Technol. Health Care.

[14] Pinho T., Tavares P., Maciel P., Pollmann C. (2005). Developmental absence of maxillary lateral incisors in the Portuguese population. Eur. J. Orthod..

[15] Kaba A.D., Marechaux S.C. (1989). A fourteen-year follow-up study of traumatic injuries to the permanent dentition. ASDC J. Dent. Child..

[16] Hamilton F.A., Hill F.J., Holloway P.J. (1997). An investigation of dento-alveolar trauma and its treatment in an adolescent population. Part 1: The prevalence and incidence of injuries and the extent and adequacy of treatment received. Br. Dent. J..

[17] Cawood J.I., Howell R.A. (1988). A classification of the edentulous jaws. Int. J. Oral Maxillofac. Surg..

[18] Udeabor S.E., Heselich A., Al-Maawi S., Alqahtani A.F., Sader R., Ghanaati S. (2023). Current Knowledge on the Healing of the Extraction Socket: A Narrative Review. Bioengineering.

[19] Tan W.L., Wong T.L., Wong M.C., Lang N.P. (2012). A systematic review of post-extractional alveolar hard and soft tissue dimensional changes in humans. Clin. Oral Implants Res..

[20] Dam V.V., Trinh H.A., Rokaya D., Trinh D.H. (2022). Bone Augmentation for Implant Placement: Recent Advances. Int. J. Dent..

[21] Jogezai U., Kalsi A. (2024). Long-term complications and management of dental trauma in the adult patient—Part 2: Discoloured, displaced and missing teeth. Br. Dent. J..

[22] Jonasson G., Skoglund I., Rythen M. (2018). The rise and fall of the alveolar process: Dependency of teeth and metabolic aspects. Arch. Oral Biol..

[23] Aghaloo T.L., Mardirosian M., Delgado B. (2017). Controversies in Implant Surgery. Oral Maxillofac. Surg. Clin. N. Am..

[24] Gotfredsen K. (2012). A 10-year prospective study of single tooth implants placed in the anterior maxilla. Clin. Implants Dent. Relat. Res..

[25] Terheyden H., Wusthoff F. (2015). Occlusal rehabilitation in patients with congenitally missing teeth-dental implants, conventional prosthetics, tooth autotransplants, and preservation of deciduous teeth—A systematic review. Int. J. Implants Dent..

[26] Park J.C., Baek W.S., Choi S.H., Cho K.S., Jung U.W. (2017). Long-term outcomes of dental implants placed in elderly patients: A retrospective clinical and radiographic analysis. Clin. Oral Implants Res..

[27] Grant B.T., Kraut R.A. (2007). Dental implants in geriatric patients: A retrospective study of 47 cases. Implants Dent..

[28] Jan Tetsch F.T. (2020). Prospective Implant Placement Following Traumatic Loss of Anterior Teeth in a Growing patient and Anticipation of Residual Growth. Int. J. Dent. Oral Health.

[29] World Medical Association (2025). World Medical Association Declaration of Helsinki: Ethical Principles for Medical Research Involving Human Participants. JAMA.

[30] Buser D., Martin W., Belser U.C. (2004). Optimizing esthetics for implant restorations in the anterior maxilla: Anatomic and surgical considerations. Int. J. Oral Maxillofac. Implants.

[31] Fürhauser R., Florescu D., Benesch T., Haas R., Mailath G., Watzek G. (2005). Evaluation of soft tissue around single-tooth implant crowns: The pink esthetic score. Clin. Oral Implants Res..

[32] Berglundh T., Armitage G., Araujo M.G., Avila-Ortiz G., Blanco J., Camargo P.M., Chen S., Cochran D., Derks J., Figuero E. (2018). Peri-implant diseases and conditions: Consensus report of workgroup 4 of the 2017 World Workshop on the Classification of Periodontal and Peri-Implant Diseases and Conditions. J. Clin. Periodontol..

[33] Bonfante E.A., Leary J., Daher S., Murcko L., Hirayama M., Bergamo E.T. (2021). Implants Placed in Adolescents Followed for Up to 15.5 Years: A Retrospective Case Series. Int. J. Oral Maxillofac. Implants.

[34] Rokn A., Bassir S.H., Rasouli Ghahroudi A.A., Kharazifard M.J., Manesheof R. (2016). Long-term Stability of Soft Tissue Esthetic Outcomes Following Conventional Single Implant Treatment in the Anterior Maxilla: 10–12 Year Results. Open Dent. J..

[35] Tanner J.M., Davies P.S. (1985). Clinical longitudinal standards for height and height velocity for North American children. J. Pediatr..

[36] Negi A., Amita (2020). Interdisciplinary management of congenitally missing maxillary lateral incisors. J. Oral Biol. Craniofac. Res..

[37] Brugnolo E., Mazzocco C., Cordioll G., Majzoub Z. (1996). Clinical and radiographic findings following placement of single-tooth implants in young patients--case reports. Int. J. Periodontics Restor. Dent..

[38] Wittneben J.G., Igarashi K., Bragger U., Daniel B., Schimmel M., Wismeijer D. (2022). Vertical eruption of anterior maxillary teeth adjacent to single-implant-supported crowns: An assessment after a 3-year follow-up period. J. Prosthet. Dent..

[39] Padovezi I., Peruzzo D., Fernandes J.C.H., Fernandes G.V.O., Joly J.C. (2025). Long-Term Assessment (5- to 19-Year Follow-up) of the Incisal- Level Changes in Single Implants Placed in the Anterior Maxilla: An Observational Clinical Study. Int. J. Oral Maxillofac. Implants.

[40] Cocchetto R., Pradies G., Celletti R., Canullo L. (2019). Continuous craniofacial growth in adult patients treated with dental implants in the anterior maxilla. Clin. Implants Dent. Relat. Res..

[41] Sauvin G., Nurdin N., Bischof M., Kiliaridis S. (2022). Assessment and aesthetic impact of a long-term vertical discrepancy between the single anterior maxillary implant-supported crown and adjacent teeth: A retrospective cross-sectional study. Clin. Exp. Dent. Res..

[42] Wittneben J.G., Molinero-Mourelle P., Hamilton A., Alnasser M., Obermaier B., Morton D., Gallucci G.O., Wismeijer D. (2023). Clinical performance of immediately placed and immediately loaded single implants in the esthetic zone: A systematic review and meta-analysis. Clin. Oral Implants Res..

[43] Wallin Bengtsson V., Lindahl C., Scholander S. (2025). Patient-reported outcomes of esthetics, function and oral hygiene with single dental implants 10–15 years after placement: A cross-sectional study. Acta Odontol. Scand..

[44] Al Najam Y., Tahmaseb A., Wiryasaputra D., Wolvius E., Dhamo B. (2021). Outcomes of dental implants in young patients with congenital versus non-congenital missing teeth. Int. J. Implants Dent..

